# Levels and distribution of self-rated health in the Kazakh population: results from the Kazakhstan household health survey 2012

**DOI:** 10.1186/1471-2458-14-768

**Published:** 2014-07-30

**Authors:** Adil Supiyev, Talgat Nurgozhin, Zhaxybay Zhumadilov, Almaz Sharman, Michael Marmot, Martin Bobak

**Affiliations:** Centre for Life Sciences, Nazarbayev University, 53 Kabanbay batyr ave., Astana, 010000 Kazakhstan; Department of Epidemiology and Public Health, University College London, London, UK; Academy of Preventive Medicine, Almaty, Kazakhstan

**Keywords:** Self-rated health, Socioeconomic factors, Central Asian countries

## Abstract

**Background:**

The high and fluctuating mortality and rising health inequalities in post-Soviet countries have attracted considerable attention. However, there are very few individual-level data on distribution of health outcomes in Central Asian countries of the former Soviet Union. We analysed socioeconomic predictors of two self-rated health outcomes in a national survey in Kazakhstan.

**Methods:**

We used data from the 2012 Kazakhstan Household Health Survey on 12,560 respondents aged 15+. Self-rated health, self-reported worsening of health, and a range of socio-demographic variables were collected in an interview. The self-rated health outcomes were dichotomized and logistic regression was used to estimate their associations with education, income, ownership of a car, second house and computer, marital status, ethnicity and urban/rural residence.

**Results:**

The prevalence of poor/very poor self-rated health was 5.3%, and 11.0% of participants reported worse health compared to 1 year ago. After controlling for age, sex and region, all socio-demographic factors were related to self-rated health. After adjusting for all variables, education and car ownership showed the most consistent effects; the odds ratio of poor health and worsening of health were 0.43 (95% confidence interval 0.32-0.58) and 0.54 (0.44-0.68) for university vs. primary education, respectively, and 0.64 (0.51-0.82) and 0.68 (0.58-0.80) for car ownership, respectively. Unmarried persons, ethnic Russians and urban residents also had increased prevalence of poor health in multivariable models.

**Conclusions:**

Despite the limitations of using subjective health measures, these data suggest strong associations between two measures of self-rated health and a number of socioeconomic characteristics. Future studies and health policy initiatives in Kazakhstan and other Central Asian countries should take social determinants of health into account.

## Background

The high mortality rates and dramatic changes in countries of the former Soviet Union since 1990 have attracted considerable attention [1–5]. In addition to the high and fluctuating mortality, the growing social disparities have been one of the dominant features of the post-communist transition. Indeed, rising socioeconomic inequalities in health have been reported from all countries where they have been studied [6–9]. However, most information to date came from Russia and the Baltic states; by contrast, very little data are available in the Central Asian republics of the former Soviet Union.

Kazakhstan has one of the highest mortality rates in the WHO European region; in 2010, life expectancy at birth (men and women combined) was 68.6 years, similar to 69.0 years in Russia but considerably lower than 81.2 years in the “old” European Union countries (WHO Health for all database). As in other post-communist countries, the transition had its winners and losers, and one would expect that those in lower socioeconomic position would have worse health. However, very little is known about the distribution of health in the Kazakh population by socioeconomic and demographic characteristics. We are aware of only one study in the literature exploring individual-level influences on health in Kazakhstan [10] and were are not aware of any published study in other Central Asian countries.

In this paper, we seek to fill this gap in the literature. We used data from the 2012 Kazakhstan Household Health Survey to investigate the associations of two self-reported outcomes (self-rated health and worsening of health with a range of socioeconomic characteristics and to assess whether these associations are independent from other socio-demographic measures. Self-rated health is a widely used health status indicator which has been shown to be a good predictor of objective health outcomes, including mortality, in studies within populations [11]. Although subjective, self-reported outcomes have been found useful in the absence of objective health measures.

## Methods

### Data source

We used the data from Kazakhstan Household Health Survey (HHS) conducted in 2012. A nationally representative multi-stage sampling approach was used. First, 452 Census Control Areas (CCA, mean size 1200 inhabitants per area) were randomly selected from complete list of all CCAs covering the entire country, proportionally to size of their respective Oblast (region). Second, 12,560 households were randomly selected from the chosen CCAs. The target population of the survey were all residential households with at least one resident aged 15 years and over. In each household, one respondent completed the survey. The response rate was 93%. Data were collected by trained interviewers using computer assisted personal interviews (CAPI).

### Measurements

We used two outcome variables. First, self-rated health was assessed by the question “how would you rate your health in general?” with responses on a 5-point scale: “very good”; “good”; “neither good or bad”; “bad”; and “very bad”. For our analyses, responses bad and very bad were combined into poor health, and responses very good, good and neither good or bad were combined into “not bad health”.

Second, in order to assess the consistency of the results, we also used a second self-rated health variable, based on the question “how would you rate your health as a whole in contrast with 1 year ago?”, again with responses on a 5-point scale: much better; better; the same; worse; much worse. The responses were dichotomized in worse (combining bad and very bad) vs. not worse (much better, better, the same).

### Socio-demographic characteristics

In addition to age and sex, a number of socio-demographic characteristics were used in the following categories. Marital status was grouped into married vs. unmarried (combining widowed, divorced and single). Education was classified into primary or less; vocational; secondary; and university. The ethnicity of respondents was categorized as Kazakhs, Russians and others. Household income was assessed by a single question on total monthly household income. The reported values were equalized for the household size (using coefficient 0.4 for the second and every other household member), and collapsed into quartiles in the final analytical sample; given the large number of missing values, persons who did not report income were classified in separate category labeled “missing income”. Finally, we used ownership of car, computer and second house as markers of the material conditions of the respondent’s household.

### Statistical analysis

We conducted a complete case analysis; i.e. we excluded 105 subjects with missing data on any of the variables used in the analysis. In descriptive analysis, we tabulated unadjusted frequencies of both health outcomes by each predictor variable. The associations between self-rated health and socio-demographic characteristics were estimated in logistic regression in 3 steps. First, we estimate odds ratios (and 95% confidence intervals) adjusted for age (5-year age groups were used a categorical variable), sex and region. Second, we additionally adjusted for education and income. Finally, all variables were included in the model simultaneously. All analyses were performed using STATA software, version 12 (Station College, Texas, USA).

## Results

The overall prevalence of poor health was 5.3%, and 11.0% reported worse health compared to 1 year ago (Table 1). The distribution of the responses on self-rated health was as follows: very good 10.2%, good 51.7%, neither good nor bad 32.7%, bad 4.8%, very bad 0.5% (not shown in table). The prevalence of both outcomes declined with increasing education and income, and it was higher among women, unmarried subjects, respondents of non-Kazakh ethnicity, in rural areas, and in persons who did not have car, second house or computer.

**Table 1:** **Descriptive characteristics of participants**

Characteristic		Number of subjects	% with poor health	% reporting worse health than 1 yr ago
**Total**		**12,455**	**5.3**	**11**
**Sex**	Men	5,198	3.5	7.7
	Women	7,257	6.5	13.5
**Age group**	15-24	1,860	0.6	1.7
	25-34	2,530	1.0	3.0
	35-44	2,280	1.4	5.9
	45-54	2,181	3.1	8.0
	55-64	1,753	7.8	18.4
	65+	1,851	20.6	34.3
**Education**	Primary or less	1,272	17.4	27.1
	Vocational	3,344	5.1	12.1
	Secondary	3,854	4.6	9.5
	University	3,985	2.2	6.5
**Income quartile**	Q1 (lowest)	2,647	6.6	12.8
	Q2	2,597	7.2	14.3
	Q3	2,680	4.7	11.1
	Q4 (highest)	2,546	3.3	6.7
	Missing	1,985	4.3	9.9
**Married**	Unmarried	5,324	7.7	14.4
	Married	7,131	3.5	8.6
**Ethnicity**	Kazakh	7,468	3.2	8.5
	Russia	3,823	9.1	15.5
	Other	1,164	5.9	13.0
**Urban**	Urban	5,016	4.6	10.4
	Rural	7,439	5.7	11.5
**Car ownership**	No	6,790	7.9	15.2
	Yes	5,665	2.1	6.1
**Second house**	No	11,799	5.4	11.2
	Yes	656	2.7	8.2
**Computer**	No	6,997	7.7	14.3
	Yes	5,458	2.1	6.9
**Region**	Almaty City	853	3.8	14.9
	Astana	1,072	1.9	4.1
	Center	976	2.3	4.9
	East	1,096	11.0	18.2
	North	2,231	7.4	15.5
	South	4,334	5.0	9.8
	West	1,893	4.2	9.8

Table 2 shows the results of logistic regression analysis of self-rated health. After controlling for age, sex and region (Model 1), all socioeconomic variables except second house were significantly associated with poor health. When education and income were added (Model 2), the effect of income was attenuated but the remaining associations did not change substantially. With all variables included (Model 3), education and car ownership remained strongly and significantly associated with poor health but the associations with marital status, Russian ethnicity and rural residence were attenuated but remained significant. However, the association with income and computer ownership became statistically insignificant.

**Table 2:** **Odds ratios (95**% **confidence intervals) of poor self-rated health by socioeconomic characteristics**

Characteristic		Model 1	Model 2	Model 3
		OR (95% CI)	OR (95% CI)	OR (95% CI)
**Education**	Primary or less	1.0	1.0	1.0
	Vocational	0.58 (0.46-0.73)	0.56 (0.44-0.71)	0.58 (0.45-0.73)
	Secondary	0.51 (0.40-0.64)	0.55 (0.43-0.69)	0.54 (0.42-0.69)
	University	0.36 (0.27-0.47)	0.42 (0.31-0.56)	0.43 (0.32-0.58)
**Income quartile**	Q1 (lowest)	1.0	1.0	1.0
	Q2	0.77 (0.61-0.98)	0.81 (0.64-1.03)	0.78 (0.61-0.99)
	Q3	0.66 (0.51-0.86)	0.73 (0.55-0.94)	0.81 (0.62-1.05)
	Q4 (highest)	0.63 (0.47-0.85)	0.72 (0.53-0.98)	0.85 (0.62-1.18)
	Missing	0.90 (0.67-1.21)	0.95 (0.71-1.28)	1.00 (0.74-1.35)
**Married**	Married	1.0	1.0	1.0
	Unmarried	1.70 (1.41-2.06)	1.55 (1.28-1.89)	1.38 (1.13-1.69)
**Ethnicity**	Kazakh	1.0	1.0	1.0
	Russia	1.44 (1.18-1.75)	1.49 (1.22-1.82)	1.29 (1.05-1.59)
	Other	1.11 (0.82-1.5)	1.06 (0.78-1.43)	1.00 (0.74-1.36)
**Urban**	Urban	1.0	1.0	1.0
	Rural	1.28 (1.06-1.54)	1.47 (1.2-1.78)	1.28 (1.05-1.56)
**Car ownership**	No	1.0	1.0	1.0
	Yes	0.52 (0.42-0.65)	0.55 (0.44-0.68)	0.64 (0.51-0.82)
**Second house**	No	1.0	1.0	1.0
	Yes	0.71 (0.43-1.17)	0.82 (0.50-1.35)	1.06 (0.64-1.77)
**Computer**	No	1.0	1.0	1.0
	Yes	0.64 (0.51-0.80)	0.71 (0.56-0.90)	0.81 (0.63-1.04)

Model 1: adjusted for age, sex and region; Model 2: additionally adjusted for income and education; Model 3: adjusted for all variables in the table.

Table 3 shows analyses of worsening of health over the last year. The results were broadly similar to those of self-rated health. Education and car ownership were very strong predictors of health worsening and the strength of the associations was not attenuated by adjustment for other characteristics. The effects of income, marital status and rural residence were attenuated but retained some of their effect. In contrast to self-rated health, ethnicity was not associated with reporting worse health than a year ago.

**Table 3:** **Odds ratios (95**% **confidence intervals) of worse health than a year ago by socioeconomic characteristics**

Characteristic		Model 1	Model 2	Model 3
		OR (95% CI)	OR (95% CI)	OR (95% CI)
**Education**	Primary or less	1.0	1.0	1.0
	Vocational	0.73 (0.60-0.88)	0.73 (0.60-0.88)	0.75 (0.62-0.91)
	Secondary	0.52 (0.43-0.63)	0.53 (0.44-0.65)	0.54 (0.45-0.66)
	University	0.52 (0.42-0.63)	0.55 (0.45-0.68)	0.54 (0.44-0.68)
**Income quartile**	Q1 (lowest)	1.0	1.0	1.0
	Q2	0.84 (0.70-1.00)	0.88 (0.74-1.06)	0.87 (0.72-1.04)
	Q3	0.82 (0.68-0.99)	0.90 (0.74-1.08)	0.93 (0.77-1.13)
	Q4 (highest)	0.59 (0.47-0.73)	0.66 (0.53-0.83)	0.71 (0.56-0.90)
	Missing	0.85 (0.68-1.05)	0.90 (0.72-1.11)	0.92 (0.73-1.14)
**Married**	Married	1.0	1.0	1.0
	Unmarried	1.42 (1.24-1.62)	1.31 (1.14-1.50)	1.22 (1.06-1.41)
**Ethnicity**	Kazakh	1.0	1.0	1.0
	Russia	0.93 (0.81-1.08)	0.96 (0.83-1.11)	0.89 (0.77-1.04)
	Other	0.97 (0.78-1.19)	0.92 (0.74-1.14)	0.90 (0.73-1.12)
**Urban**	Urban	1.0	1.0	1.0
	Rural	1.08 (0.95-1.24)	1.21 (1.05-1.39)	1.13 (0.98-1.31)
**Car ownership**	No	1.0	1.0	1.0
	Yes	0.66 (0.57-0.75)	0.69 (0.60-.80)	0.68 (0.58-0.80)
**Second house**	No	1.0	1.0	1.0
	Yes	0.96 (0.71-1.31)	1.11 (0.81-1.51)	1.19 (0.87-1.63)
**Computer**	No	1.0	1.0	1.0
	Yes	0.96 (0.83-1.10)	1.09 (0.93-1.26)	1.20 (1.02-1.40)

Model 1: adjusted for age, sex and region; Model 2: additionally adjusted for income and education; Model 3: adjusted for all variables in the table.

## Discussion

In this large national survey in Kazakhstan, we found strong associations between two measures of self-rated health and a number of socioeconomic characteristics. Education and car ownership showed the strongest and most consistent associations with self-rated health. The effects of income were attenuated in multivariable models. Russian ethnicity, unmarried status and rural residence were also associated with self-rated health, but their effects were less consistent between the two health measures.

The major limitation of this study is the cross-sectional nature of the data. As socioeconomic position may be influenced by declining health, it is difficult to be confident about the direction of the associations. While education is usually completed relatively early in life and it is unlikely to be affected by health status, we can be less certain that income and ownership of car, second house or computer indeed preceded changes in health.

The second major limitation is the fact that both our health outcomes were self-reported. However, self-reported health is an important outcome; there is an extensive evidence that self-reported health is a useful indicator of health, and it strongly predicts mortality and other outcomes, including cardiovascular disease and cancer [11–14], although caution is needed when extrapolating results from self-rated health to mortality [15]. Self-reported measures may not be reliable in cross-cultural comparisons [16], and cultural patterns in responding to questions may affect the absolute levels of self-rated health. The levels of poor health observed in this study were substantial lower than in other former communist countries [17, 18]. As this study was organised by the Kazakh Ministry of Health, the interviewers may have been perceived as governmental officials; this may have contributed, in addition to cultural norms, to a positive mode of reporting. Although the two questions on self-reported health used in our analyses would partly reflect different influences (the question on health compared to a year ago would be more likely to reflect changes over the past year), the general pattern of results was consistent, supporting the validity of the outcome.

An important methodological issue to consider is the generalizability of the results. The HHS sample was designed to be nationally representative, but this aim can be affected by incomplete sampling frame, imperfect identification of samples households, or non-response. We are not aware of problems with the sampling frame or fieldwork and response rate was high. Non-responders generally tend to be less healthy, have less favourable health behaviours, and have less privileged socioeconomic position. All this could affect the absolute levels of health and socioeconomic indicators in the sample. As mentioned above, the prevalence of poor health in HHS was lower than in other post-communist countries, and this might to some extent be due to the high response rate (leading to inclusion of more healthy persons than at lower response rates). In any case, however, response rate is unlikely to bias the estimates of associations between variables.

Despite these limitations, our study provides useful evidence, particularly given the lack of individual-level data from the Central Asian countries of the former Soviet Union. The countries in this region followed heterogeneous patterns of socioeconomic development after the dissolution of Soviet Union, with different countries choosing different health or social systems [19, 20]. However, many of the uniform challenges are similar, including the rising social inequalities [21, 22].

The life expectancy in Kazakhstan follows trends similar to other Central Asian countries but considering that its GDP per capita is the highest in Central Asia ($13,900 in 2012), life expectancy in Kazakhstan is surprisingly low. Kazakhstan has high mortality from CVD and from external causes but we are not aware about any data on socioeconomic distribution of these important conditions within the Kazakh population (or any other Central Asian country).

Consistently with findings in a recent report from survey in Almaty [10], our results on self-rated health suggest considerable socioeconomic differentials in health in Kazakhstan. In crude analyses, differences were present by virtually all socioeconomic characteristics available in the survey. However, the multivariate analyses revealed several interesting features. Firstly, education was the most robust and consistent predictor of both health outcomes. This is consistent with other research in a number of post-communist countries [23–27]. Since education is unlikely to be affected by the reverse causation bias, it remains the most useful and readily available indicator of individual socioeconomic position in former communist countries. In most populations with available data, education is associated with health behaviours (associated with chronic diseases), health care seeking behaviours and mental health, all of which are likely to be associated with self-reported health. In addition, as education is completed relatively early in life, it is also likely to reflect accumulation of health insults over the life course. The fact that education was not substantially affected by adjustment for income may suggest that the influences linked to education are more important than income or factors associated with income.

Income has often been used as the primary marker of socioeconomic position in some western-based studies. In our data from Kazakhstan, it was strongly related to both health outcomes but the association was substantially attenuated in multivariable models. In fact, car ownership seemed to perform as good as, or better than, income in predicting health. This may be due to misreporting or misclassification of income by study participants, as a single question is often considered inadequate to measure income precisely. On the other hand, car ownership in Kazakhstan may have a particular significance, potentially being a visible marker of socioeconomic position (as long as the poor performance of the income variable was not entirely due to income misclassification) or a measure of wealth (of which income is not a good measure). In addition, the association of income with self-reported health was substantially attenuated by education, suggesting that education may act as a confounder for income. In either case, car ownership is measured more easily and more precisely in population-based studies, and it may be a more practical measure of material conditions for population studies in the region.

By contrast, ownership of second house was not an important predictor of either health outcome. This is probably due to the fact “second house” may have different meanings – from a dacha (not a specific measure of wealth) to second apartment in a city (which, anecdotally, is a measure of wealth). Similarly, computer ownership was not statistically associated with health in multivariable models; this may reflect the fact that computers became widely affordable and are not anymore marker of wealth.

We found that participants reporting Russian ethnicity had moderately increased risk of poor health. This pattern is consistent with many other studies in former Soviet countries. It may reflect either genuinely worse health status of the Russian minorities, consistent, for example, with higher mortality of Russians in census-linked analyses in Lithuania [28] or unlinked data from Kyrgyzstan [29]. Alternatively, however, we cannot exclude the possibility of differential reporting of self-rated health in Kazakh Russians compared to other Kazakh ethnicities.

We found slightly worse health in urban population than in rural areas. This is surprising, given the higher income and education in urban areas. We can only speculate whether the difference may be due to differential reporting between rural and urban respondents, perhaps due to differential expectations. On the other hand, it is reassuring that, consistently with most other studies, poor health was associated with unmarried status and female sex.

## Conclusion

Overall, data from the Kazakh national study have shown considerable differentials in self-rated health by several socioeconomic characteristics. The results are broadly consistent with studies in western European and in other eastern European countries. Education and car ownerships were the best predictors of health. The results suggest that, as in other parts of the world [30], socioeconomic factors are powerful determinants of health in Central Asian countries, and governments should pay attention to social inequalities in health as part of their policies.
